# Bayesian adjustment for measurement error in continuous exposures in an individually matched case-control study

**DOI:** 10.1186/1471-2288-11-67

**Published:** 2011-05-14

**Authors:** Gabriela Espino-Hernandez, Paul Gustafson, Igor Burstyn

**Affiliations:** 1 Department of Statistics, University of British Columbia, Vancouver, BC, Canada; 2 Department of Environmental and Occupational Health, School of Public Health, Drexel University, Philadelphia, PA, USA

## Abstract

**Background:**

In epidemiological studies explanatory variables are frequently subject to measurement error. The aim of this paper is to develop a Bayesian method to correct for measurement error in multiple continuous exposures in individually matched case-control studies. This is a topic that has not been widely investigated. The new method is illustrated using data from an individually matched case-control study of the association between thyroid hormone levels during pregnancy and exposure to perfluorinated acids. The objective of the motivating study was to examine the risk of maternal hypothyroxinemia due to exposure to three perfluorinated acids measured on a continuous scale. Results from the proposed method are compared with those obtained from a *naive *analysis.

**Methods:**

Using a Bayesian approach, the developed method considers a classical measurement error model for the exposures, as well as the conditional logistic regression likelihood as the disease model, together with a random-effect exposure model. Proper and diffuse prior distributions are assigned, and results from a quality control experiment are used to estimate the perfluorinated acids' measurement error variability. As a result, posterior distributions and 95% credible intervals of the odds ratios are computed. A sensitivity analysis of method's performance in this particular application with different measurement error variability was performed.

**Results:**

The proposed Bayesian method to correct for measurement error is feasible and can be implemented using statistical software. For the study on perfluorinated acids, a comparison of the inferences which are corrected for measurement error to those which ignore it indicates that little adjustment is manifested for the level of measurement error actually exhibited in the exposures. Nevertheless, a sensitivity analysis shows that more substantial adjustments arise if larger measurement errors are assumed.

**Conclusions:**

In individually matched case-control studies, the use of conditional logistic regression likelihood as a disease model in the presence of measurement error in multiple continuous exposures can be justified by having a random-effect exposure model. The proposed method can be successfully implemented in WinBUGS to correct individually matched case-control studies for several mismeasured continuous exposures under a classical measurement error model.

## Background

Measurement error refers to the variation of the observed measurement from the true value, and consists of two components, random error and systematic error. The first component, the random error, is caused by any factors that randomly affect the measurement across a sample, and usually arises from inaccuracy in a measuring laboratory instrument or random fluctuations in the environmental conditions. The second error component, the systematic error, is caused by any factors that systematically affect the measurement across a sample, and can be attributed to non-random problems in the system of measurement (e.g. wrong use or improper calibration of the measurement instrument).

In many scientific areas where statistical analysis is performed, the problem of dealing with explanatory variables subject to measurement error is present. In particular, in epidemiologic studies, the explanatory variables (or 'exposures') that reflect exposure to suspected risk factors associated with a disease (the outcome variable) are commonly measured with error. These errors can be either *differential *or *non-differential*, according to whether they depend on the values of other variables in the study, for instance the outcome variable [1,2]. As has been discussed by many authors [3-6], measurement error reduces power for detecting relationship between exposures and disease, and ignoring this error may bias the assessment of the association between health outcome and exposure variables. In particular, ordinary logistic regression can lead to biased estimates of odds radios (ORs) when the covariates are subject to measurement error [7]. Researchers have proposed non-Bayesian methods to correct for measurement error in exposures in individually matched case-control studies. For instance, Guolo et al. [8] used conditional likelihood methods to correct for measurement error in a single continuous exposure using simulated data. These authors compared the performance of the likelihood methods with two other corrections techniques (regression calibration [7] and simulation-extrapolation (SIMEX) [9]), observing that the likelihood approach outperforms the alternative methods when a single continuous exposure is measured with error. McShane et al. [5] proposed a conditional scores procedure to correct for measurement error in some components of one or more continuous covariates. In that study, the authors treated the true covariates as fixed unknown parameters, which were removed from the likelihood by conditioning on a sufficient statistic and estimated together with the unknown parameters. However, the conditional scores procedure experienced convergence problems in the presence of large relative risks or when large measurement errors were considered. Also, conditional scores procedures are typically not very generalizable when data structures are changed even slightly. In addition, Liu et al. [6], Prescott and Garthwaite [10], and Rice [11] proposed Bayesian adjustments for misclassification of a binary exposure variable. Nevertheless, to our knowledge, very little attention has been given to measurement error in multiple continuous exposures in matched case-control studies, except for McShane et al. [5] whose procedure may be challenging numerically, and which is quite dependent on the settings of the problem.

Thus, in this paper, we develop a Bayesian method to correct for measurement error in multiple continuous exposures in individually matched case-control studies that may be generalized to different settings, where information regarding the measurement error variability is available from additional experiments. The methodology is illustrated using data from a study of association of perfluorinated acids (PFAs) with disruption of thyroid homeostasis in pregnant women [12]. PFAs are global contaminants of human blood and environment [13]. The objective of the motivating study was to examine the risk of maternal hypothyroxinemia due to exposure to three PFAs measured on a continuous scale. No human study has previously examined the influence of PFAs on the development of hypothyroxinemia, but there are reports on the relationship between PFAs and thyroid hormones and thyroid disease. In a sample from the US general population from 1999 to 2006, both men and women exposed to some PFAs had higher prevalence of physician-diagnosed *thyroid disease *[14]. However, a small study in a highly contaminated community failed to find similar association [15], and two other studies also did not report associations [16,17]. Dallaire et al. [18] reported a mixture of negative and positive associations of thyroid hormones with PFAs. The extent to which measurement error may contribute to apparent heterogeneity among these reports is unknown, but it certainly should be considered as an explanation.

We start this paper by describing the data in the motivating example in detail, followed by derivation of an estimate of the random error variability from *percent recovery *experiments, description of the proposed Bayesian model and justification of conditional logistic disease model for measurement error correction. Next, application of the method is illustrated along with a sensitivity analysis of the impact on the results if greater-than-estimated random error was present. The proposed Bayesian method is implemented in WinBUGS software and inferences are compared to those drawn from a *naive *analysis, which ignores measurement errors in the exposures.

## Methods

### Data

The developed Bayesian method is illustrated using individually matched case-control data from a study of Chan et al. [12]. The objective of the study was to examine the risk of maternal hypothyroxinemia due to exposure to three PFAs: perfluorooctanoic acid (PFOA), perfluorooctane sulfonate (PFOS) and perfluorohexane sulfonate (PFHxS). Chan et al. [12] extracted PFAs from maternal sera samples from 271 pregnant women, aged 18 or older, who elected to undergo a second trimester prenatal "triple screen", delivered at 22 weeks gestation or more to live singletons without evidence of malformations, and were referred by a physician who made a least eight recommendations for the "triple screen" over the study period. The exposure variables were reported on a continuous scale, and censored/non-detectable values (about 5.4% of the total number of records) were recorded as half the value of the limit of detection. Concentrations of the PFAs were transformed to log-molar units, and it was seen that after this transformation the measured exposures approximately follow a normal distribution. A quality control experiment was performed in order to assess the amount of error incurred in the measurement of the exposures. In this experiment, percentages of recovery were calculated for each exposure and the results revealed the presence of a random error in the measurements. Details of this procedure and results are presented in Appendix I.

Chan et al. [12] classified the subjects into cases or controls, based on the analysis of their thyroid stimulating hormone (TSH) and free thyroxin (T4) concentrations. The hypothyroxinemia cases correspond to women exhibiting normal TSH concentrations with no evidence of hyperthyroidism (between 0.15 and 4.0 mU/L) and free T4 in the 10^th ^percentile (less than 8.8 pmol/L). Meanwhile the controls correspond to women with normal TSH concentrations but having free T4 concentrations between the 50^th ^and 90^th ^percentiles (between 12.0 and 14.1 pmol/L). Each case was matched to between one and three controls on the basis of two matching factors: maternal age at blood draw (± 3 years) and referring physician (a total of 29 physicians). Further details on the construction of the data can be found in Chan et al. [12].

In summary, the matched case-control data used to illustrate the Bayesian method to correct for measurement error contain information from 96 cases and 175 individually matched controls. For the purpose of this paper, it is assumed there is no misclassification of control/case status. In addition, the data contain, for each subject, the corresponding exposure to PFOA, PFOS and PFHxS, which are reported on a continuous scale in log- molar units and are assumed to be subject only to random measurement error. Moreover, four potential confounders which are precisely measured are reported: maternal age (years), maternal weight (pounds), maternal race (Caucasian and non-Caucasian) and gestational age (days). All potential confounders except for maternal race were reported on a continuous scale. The maternal age variable is retained despite its use as a matching factor, in case the matching is too coarse to fully eliminate confounding.

### Measurement model

Generally, in observational studies, the vector of imprecise surrogate exposures *W* is commonly recorded, instead of *X* itself. Therefore, in order to understand the relationship between the disease risk and the explanatory variables *X*, having data on *Y *and *W*, it is necessary to account for measurement error in the exposures. In this paper, the attention is concentrated on the problem of having only random error, by assuming zero systematic error. However, the present methodology can be adapted to introduce the effect of a systematic error.

Assume the vector of independent surrogates *W* arises from a classical additive measurement error model, which can be expressed as(1)

where *U* refers to the measurement error component. This classical model assumes the true exposures are recorded with an additive, independent error. In addition, it can be assumed the measurement error is *non-differential*, and *unbiased*. The assumption of *non-differential *measurement error refers to the fact that the distribution of the surrogate exposures depends only on the actual exposure variables and not on the response variable or other variables in the model. As a result, the conditional distribution of (*W*|*X*,*Y*) is identical to the conditional distribution of (*W*|*X*). The *unbiased *assumption *E*(*U*|*X*) = 0 implies *E*(*U*|*X*) = *X*. Typically, the measurement error component is also assumed to be normally distributed with constant variance, i.e. *U *~ *N*_*P*_(0, *∑*), where is *∑ *a diagonal matrix with the main diagonal entries given by , for *p *= 1, ..., *P*.

Under the stated assumptions, *W*|*X* follows a *P*-dimensional multivariate normal distribution with a mean vector given by the vector of true exposures and a covariance matrix *∑*, which in this case is known. Thus, the density corresponding to the measurement model is given by *W*|*X*, *∑* ~ *N*_*p*_(*X*, *∑*). Therefore, under the assumptions of an individually matched case-control data, the density of the measurement model is given by(2)

where *w*_*ij *_and *x*_*ij *_correspond to the vector of surrogate and true exposures variables, respectively, for the *j *- *th *member of the *i *- *th *matched set, and *N *refers to the number of matched sets.

For the particular case of data used in the study on PFAs, the surrogate variables are measured concentrations of PFOA, PFOS, and PFHxS, which correspond to the exposures to the compounds reported on a continuous scale in log-molar units. Consequently, an additive measurement error model for the exposures in log-molar units translates into a multiplicative error structure, in which the corresponding error term is proportional to the true exposure in molar scale. In many epidemiological studies, positive explanatory variables are subject to this sort of measurement error. Using available validation data from the quality control procedure performed by Chan et al.[12], the covariance matrix *∑* of the measurement model can be estimated. In Appendix I we present a statistical argument for estimating *∑* from this particular form of quality control data. The argument is based on the multivariate version of the delta method [19] and uses the estimated standard deviation of the percentages of recovery for the concentrations of the three compounds in parts-per-billion to obtain information about the incurred error in the measurement of the exposures.

### Disease model

In order to describe a relationship between the true exposures and the probability associated to the response variable, it is necessary to specify a disease model. Since the study analysed in this paper involves matched sets, the conditional logistic regression likelihood is adopted.

Consider a study having *N *matched sets, such that the *j-th *member (*j *= 1, ..., *n*_*i*_) of the *i-th *set (*i *= 1, ..., *N*) has *P *associated continuous exposures *X*_*ij *_= (*X*_*ij*1_, ..., *X*_*ijp*_)^*T*^. In addition, let *Y*_*i *_= (*Y*_*i*1_, ..., *Y*_*ini*_)^*T *^be a vector of response variables associated to the *i-th *matched set, such that *Y*_*ij *_= 1 for the cases and *Y*_*ij *_= 0 for the controls. Without loss of generality, the subjects can be labelled such that *Y*_*i*1 _= 1 and *Y*_*ij *_= 0, for *j *= 2, ..., *n*_*i*_. Thus, the underlying objective is to model the retrospective probabilities for the case (i.e. *P*(*X*_*i*1 _| *Y*_*i*1 _= 1) ), and the controls (i.e. *P*(*X*_*ij *_| *Y*_*ij *_= 0), for *j *= 2, ... *n*_*i*_), which can be accomplished by using the conditional logistic regression model.

The conditional likelihood is obtained by conditioning on the number of cases in each matched set, i.e. conditioning on . In the particular case of individual matching, the number of cases is one; therefore, the conditional likelihood for the *i *- *th *matched set (*i *= 1, ..., *N*) is given by [20,21]

where *β*= (β_1_, ..., β_*P*_)^*T *^corresponds to the log ORs associated with unit changes in each of the exposures. Under the assumptions of a matched case-control study, the full conditional likelihood is the product of  over the *N *strata or matched sets, which is(3)

The parameter *β*is assumed to be constant across matched sets, and it is the target of statistical inference.

### Bayesian model

Consider a retrospectively collected matched case-control data where each case is matched to one or more controls based on suspected confounders as matching factors. Let *Y *be the response variable, such that *Y *= 1 for cases and *Y *= 0 for controls, let *X*= (*X*_1_, ..., *X*_*P*_)^*T *^be the *P *-dimensional vector of the true, latent, continuous exposures which are subject to measurement error, and let *W*= (*W*_1_, ..., *W*_*P*_)^*T *^be the *P*-dimensional vector of surrogate exposures.

The aim of this subsection is to develop a Bayesian method to understand the association between the vector of continuous exposures *X* and the probability of the response variable *Y*, after correcting for random measurement error in the exposures.

Under the Bayesian paradigm, the posterior density of the unknown quantities is given by(4)

where θ refers to the vector of unknown parameters. The first term of the right hand side of (4) refers to the joint posterior distribution of the true exposures *X* and the surrogate variables *W*. As will be shown in Appendix II, this term contains the densities of the measurement model, disease model and exposure model. Meanwhile, the second term corresponds to the prior distribution of the unknown parameters.

### Exposure model

The conditional logistic regression model has been successfully applied in matched retrospective case-control studies, and the use of this procedure has been statistically justified using Bayesian (see for example [11,22,23]) and non-Bayesian (see for instance [20,21]) approaches. This justification is based on the fact that the likelihood term describing the distribution of the total number of cases within-stratum given the exposures can be discarded. The reason for this is that it does not provide information about the parameter of interest, since the likelihood is only a function of the unknown parameter *β*. However, this justification is no longer directly applicable when adjusting for measurement error in exposures, since the omitted likelihood term might also contain information about these exposures [6], i.e., the likelihood is a function of *β *and the unobserved exposures. As a result, the use of a conditional likelihood approach in the presence of measurement error in multiple continuous exposures has not been widely adopted.

We justify the use of conditional logistic regression likelihood as a disease model when adjusting for measurement error in an individually matched case-control study via a random-effect exposure model; details are presented in Appendix II. A different approach that does not involve a random-effect exposure model is provided by Gulo et al. [8].

In order to describe the random-effect exposure model, we assume that the vector of exposures for the *j *- *th *subject from the *i *- *th *matched set follows a multivariate normal distribution around the vector of exposure means of the corresponding matched set. Moreover, since the vector of exposure means of a matched set is unknown, we assume that this vector follows a multivariate normal distribution centered on the across-set exposures means. That results in

and

where , and , such that *γ*_*ij *_and *λ*_*i *_are mutually independent. Also notice that the within-stratum covariance matrix *V*_*W *_is assumed to be constant across matched sets. As a result, , and . Thus, the density corresponding to the random-effect exposure model is given by(5)

It has been assumed that the vector of true exposures follows a *P*-dimensional multivariate normal distribution. However, in observational studies, exposures often have a skew distribution [24]. Therefore, it is important to keep in mind that incorrect model specification may lead to biased estimates. To overcome potential misspecifications, for the univariate case some authors [24-26] have proposed the use of flexible distributions to increase the robustness to model specification. However, implementation of such methods can be quite challenging in the context of multivariate exposures.

### Joint posterior density

For the particular case of this paper, the data considered consist of *N *= 96 matched sets, such that the *i-th *matched set has *j *= 1, ..., *n*_*i *_subjects, with *n*_*i *_∈ {2,3,4}. Thus one subject is the case per set and the remaining *n*_*i *_- 1 subjects are the controls. Let  be the vector of unknown parameters. Therefore, by (4) and (AII.3), and using densities in (2), (3) and (5), it follows that the posterior density of the unknown quantities can be expressed as

It is commonly assumed that the unknown parameters are independent of each other *a priori*, so that . In order to implement a Bayes-Markov chain Monte Carlo (MCMC) inference, it is necessary to assume prior distributions for the unknown parameters. Proper prior distributions are assumed for all the parameters. Moreover, the corresponding hyperparameters are chosen so that the parameters reflect prior ignorance:

where *W*_*P*_(*R, b*) indicates a *P*-dimensional Wishart distribution with a positive definite inverse scale matrix *R*, and *b *degrees of freedom. And *I*_*P *_is an identity matrix of size *P*. For the particular case of the matched case-control data from the epidemiological study on PFAs, *P *= 3 and *μ*is estimated using the across-set sample mean of the corresponding observed exposures.

### Adjustment for additional confounders

Considering the possibility that confounding is only partially addressed by matching, further potential confounders can be introduced in the disease model. In general, potential confounders should also be included in the exposure model; however, for simplicity these confounders are not considered in our random-effect exposure model, keeping it as presented in equation (5). For the case of the PFA's data, this simplification might be justified by the fact that the exposures and the confounders exhibit small correlations (less than 0.18), so we do not expect the potential confounders to be very helpful in reconstructing the true exposures. In addition, due to the assumption of *non-differential *measurement error, the measurement model also remains as presented in equation (2).

Consider the situation where the *j-th *member of the *i-th *set has associated *K *potential confounding variables *Z*_*ij *_= (*Z*_*ij1*_, ..., *Z*_*ijk*_)^*T *^which are precisely measured. Therefore, the full conditional likelihood corresponding to the disease model in (3) can be rewritten as

where *δ*= (δ_1_, ..., δ_*K*_)^*T *^is the vector of parameters associated with the confounding effect.

Thus, the posterior density of the unknown quantities can be rewritten as

where  is the new vector of unknown parameters, and a proper and diffuse prior distribution is assumed for the parameter *δ*, by having *δ*~ *N*_*k*_(0, 10000*I*_*K*_), where *K *= 4 for the particular case of the motivating example.

## Results and Discussion

In this section, the proposed Bayesian method to correct for measurement error is illustrated using data from the study of Chan et al. [12]. Inferences drawn from a *naive *analysis and an analysis correcting for measurement error are presented. The *naive *analysis ignores error in exposure measurements, by pretending the observed exposures (PFOA, PFOS, and PFHxS) are precisely measured. Meanwhile, in the analysis accounting for measurement error, the surrogate exposures are corrected for random measurement error. Two models are considered in each analysis: a simple model assuming the only confounding is via matching factors, and a model adjusted by four further potential confounders (maternal age, maternal weight, maternal race, and gestational age). In summary, the results from four Bayesian models are compared: a simple model under the *naive *analysis (N-S), an adjusted model by confounders under a *naive *analysis (N-A), a simple model under a measurement error analysis (ME-S), and an adjusted model by confounders under a measurement error analysis (ME-A).

The models are implemented in WinBUGS software, version 1.4.3 [27], which is freely distributed and can be downloaded from the web [28]. Our WinBUGS code as available (Additional file 1). The analysis of the results was carried out using the statistical package R, version 2.11.1, which is also freely distributed on the web [29]. Two MCMC chains of length 55,000 were run for each model, using different initial values. The first 5,000 "burn-in" iterations were discarded from each chain and the last 50,000 MCMC iterations were used to perform Bayesian statistical inference. The computer running times on an Intel Core 2 Duo CPU at 2.10 GHz with 3.00 GB of RAM for N-S and N-A were approximately 1.5 and 4 minutes, respectively. Meanwhile, running times for ME-S and ME-A were about 9 and 13 minutes, respectively. The convergence to the posterior distributions and mixing of the two chains were assessed from the trace, autocorrelation, and the Gelman-Rubin convergence statistic plots. Moreover, under both types of analysis the estimated Monte Carlo standard errors of the posterior log ORs were smaller than 0.0026 for the simple models (N-S and ME-S) and smaller than 0.0030 for the models adjusted by the confounding variables (N-A and ME-A).

Posterior means and 95% equal-tailed credible intervals of the ORs obtained for the models under the *naive *Bayesian analysis and the proposed Bayesian method to correct for measurement error are depicted in Table 1. This table indicates that under the two types of analysis, there is an absence of evidence for an association between the risk of maternal hypothyroxinemia and exposure to the PFAs. The point and credible intervals estimates of the ORs under both analyses are very similar, suggesting that a slight adjustment is manifested for the level of measurement error exhibited in the PFAs. However, *a priori*, there was not intuition that the adjustment would necessarily be slight.

**Table 1:** Comparison of posterior means and credible intervals of the ORs

	OR	95% Cred. Int.	Adjusted OR	95% Cred. Int.
*Naive analysis*				

**PFOA**	**0.905**	(0.661, 1.209)	**0.828**	(0.584, 1.127)

**PFOS**	**0.802**	(0.495, 1.214)	**0.752**	(0.445, 1.181)

**PFHxS**	**1.315**	(0.964, 1.755)	**1.302**	(0.934, 1.779)

*Measurement error analysis*				

**PFOA**	**0.904**	(0.656, 1.212)	**0.821**	(0.568, 1.131)

**PFOS**	**0.794**	(0.482, 1.221)	**0.743**	(0.431, 1.191)

**PFHxS**	**1.333**	(0.960, 1.816)	**1.329**	(0.938, 1.856)

Posterior means and 95% equal-tailed credible intervals of the ORs for the simple model and the model adjusted for confounding variables. Results are presented for the Bayesian *naive *analysis and for the Bayesian method proposed to correct for measurement error.

**Figure 1:**
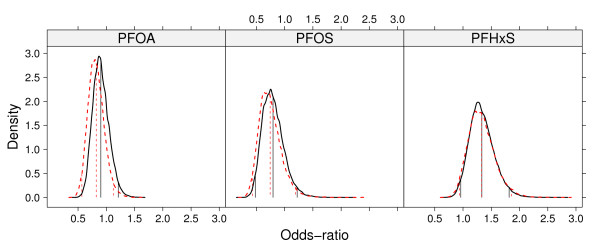
**Posterior distributions of ORs and credible intervals**. Posterior distributions (curves), and corresponding posterior means and 95% equal-tailed credible intervals (vertical lines) of ORs based on the two models under the measurement error analysis. The solid curves/lines correspond to the simple model (ME-S) and the dashed curves/lines correspond to the model adjusted for confounding variables (ME-A).

Figure 1 presents the posterior densities and 95% credible intervals of the ORs for the simple model and the model adjusted by confounders, both under the measurement error analysis. Plots indicate there is no substantial association between the exposure to any PFA and maternal hypothyroxinemia. In addition, plots show a wider posterior distribution of the OR for the exposure to PFHxS, suggesting a bigger uncertainty in the risk associated to this exposure. Moreover, the posterior distributions for the simple model and the model after adjusting for the confounding variables are quite similar, in particular for the exposures PFOS and PFHxS, suggesting little or no further confounding effect. This conclusion is confirmed after calculating 95% equal-tailed credible intervals of the estimated parameter *δ*(not shown).

**Figure 2:**
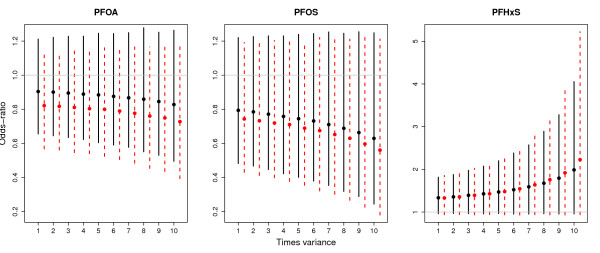
**Sensitivity analysis of measurement error variability**. Posterior means and 95% equal-tailed credible intervals of the ORs for different scenarios of measurement error variability. The solid lines correspond to the simple model (ME-S) and the dashed lines to the model adjusted for confounding variables (ME-A).

A sensitivity analysis of the measurement error variability *∑* estimated in Appendix I is carried out in order to validate the performance of the method. Figure 2 gives posterior means and 95% equal-tailed credible intervals for the ORs after increasing up to ten times the assumed measurement error variability for the exposures. Plots show that, for all the exposures, more substantial measurement error adjustments arise if larger measurement errors are assumed. Furthermore, the estimated ORs move in the anticipated directions, i.e., away from the null. However, in all cases the credible intervals widen, so that they still include the value of one, providing little evidence of any association between exposures and the outcome, regardless of the assumed measurement error magnitudes. The aforementioned MCMC diagnostics indicated that MCMC convergence and mixing worsened slightly as the assumed measurement error magnitude increased. However, chains of length 55,000 were run with the first 5,000 interactions used as a "burn-in" period were still adequate.

## Conclusions

We propose a Bayesian method to correct for measurement error in multiple continuous exposures for individually matched case-control studies. This method assumes a classical measurement model in order to account for random error in the exposures. It uses the conditional logistic regression likelihood as a disease model. We justify the use of this model in the presence of measurement error in the exposures by having a random-effect exposure model.

The proposed method can be implemented in WinBUGS software, which manages the computational complexity associated with likelihood-based approaches, to which Guolo et al. [8] referred. Moreover, as was pointed out by Guolo et al. [8], the likelihood-based methods, such as Bayesian and maximum-likelihood methods, perform well under different measurement error structures, can provide accurate inferential results, and outperform other corrections techniques (regression calibration and SIMEX). Furthermore, unlike the method proposed by McShane et al. [5] to correct for measurement error in continuous exposures, the Bayesian method proposed in this paper is neither prone to convergence errors nor highly dependent on the settings of a particular individually matched case-control study.

For the particular case of the study on PFAs, Bayesian inference of ORs indicates that little adjustment for exposure measurement error is needed for the magnitude of error determined from the quality control experiment. However, bigger adjustments arise if larger measurement errors are assumed.

Some avenues for future research are suggested by our results. First, the method assumes a multivariate normal distribution on the exposures. However, it is important to keep in mind that a model misspecification may lead to biased estimates. In this context some authors have proposed the use of parametric and non-parametric flexible models. Nevertheless, some complications are involved in their implementations. For instance, Richardson et al. [24] proposed using a normal mixture model under a Bayesian approach and found that in the absence of validation data, their approach requires very strong priors on the mixture parameters to obtain reasonable estimates. Carroll et al. [25] suggested the use of a Bayesian approach in order to avoid the complicated implementation of the Estimation-Maximization (EM) algorithm under a traditional frequentist analysis once the normal mixture model is implemented into the likelihood. Furthermore, they advised to use partially proper priors in order to avoid improper posteriors. Guolo [26] suggested the use of skew-normal family of distributions as long as this distribution is a good approximation of the distribution of the unobserved exposures in the case-control sampling. Generally, however, the implementation of flexible exposure models for multivariate exposures remains challenging.

Second, we have not made explicit comparisons between our method and other methods. We have, however, considered implementation issues for our method versus others. Particularly, we considered regression calibration techniques which impute best-guess exposure values and then plug these in to the disease model. While this is a simple procedure with some data formats, it would be no simpler that our method in the present format. The imputation involves estimating *E*(*X*|*W*), which in turn requires estimating variance components from a multivariate random effect model applied to unbalanced data, in order to acknowledge variation between and within matched sets, in a similar fashion to [5]. Thus fitting a model similar to our exposure model is required, for which software options are somewhat limited. Moreover, regression calibration requires post-fitting adjustment of standard errors, say by bootstrapping, which would be very burdensome computationally in the present setting.

Finally, using available information from the quality control experiment performed on the PFA concentrations and the multivariate version of delta method, we present a statistical approach to estimate the measurement error variability. However, different assumptions and estimation methods can be developed in the presence of additional validation data or a different structure of quality control data. For instance, the complicated structure of the percent recovery experiments necessitated a 'plug-in' approach to dealing with the measurement error covariance matrix. Simpler data structures for informing the measurement error variance, such as a validation subsample, replicates, or an instrumental variable, would much more easily lend themselves to incorporating uncertainty about this covariance matrix as part of the overall Bayesian analysis.

## Competing interests

The authors declare that they have no competing interests.

## Authors' contributions

GEH developed and implemented the Bayesian method under supervision of PG. IB designed epidemiological study, oversaw its conduct and identified the need to better understand the impact of measurement error on *naive *analysis. All authors contributed to writing of the manuscript, read its final form and approved it.

## Appendix I. Measurement error variability estimation

In reference to the epidemiological matched case-control study on PFAs, Chan et al. [12] performed a quality control procedure on the PFAs in ppb concentrations. Serum samples for subjects were divided into batches of approximately 16 per set for analysis of PFAs. Each set had a pooled sample consisting of: a paired sample of spiked serum (50 ppb of mixed standard in pooled serum) and unspiked serum (only pooled serum), besides a gold standard sample (50 ppb into methanol). Percentages of recovery were calculated by comparing the spiked concentration (i.e. difference between the paired spiked and unspiked samples) to the gold standard sample. The results showed that the standard deviations of the percent recoveries for PFAs in ppb concentrations were: 0.157 for PFOA, 0.139 for PFOS and 0.252 for PFHxS.

Let , , and  be the unspiked serum, spiked serum and gold standard corresponding to exposure *p *in ppb concentration, with *p *ε {1,2,3} = {*PFOA, PFOS, PFHxS*}. Using this notation, the percent recovery corresponding to exposure *p *is given by

Let *m*_*p *_be the factor used to convert molar units to ppb concentrations, corresponding to exposure *p*. Therefore, the percent recovery can be expressed as(AI.1)

where *W*_*p*_, *W*_*p, spiked *_and *W*_*p, gold *_correspond to the unspiked serum, spiked serum and gold standard samples in log-molar concentrations, respectively.

Using the normality and homogeneity assumptions of the error component, the corresponding equation (1) for a particular exposure *p *is equivalent to

Therefore, it follows that

By substituting these three equations into (AI.1), it is possible to see that(AI.2)

Moreover, according to the description of the samples in the quality control procedure *X*_*p,spiked*_, *X*_*p,gold*_, which correspond to the true spiked serum and gold standard samples in log-molar concentrations, have the following underlying structures(AI.3)

Substitution of (AI.3) into equation (AI.2) yields

where *c*_*p *_= *m*_p_* exp(*X*_*p*_). Therefore, the percentage of recovery corresponding to exposure *p *is a twice-differentiable function of *T*_*p *_= (ε_*p*_, ε_*p,spiked*_, ε_*p,gold*_)^*T*^. Assuming (ε_*p*_, ε_*p,spiked*_, ε_*p,gold*_) are independent, *T*_*p *_follows a trivariate normal distribution with a mean vector of zeros and a covariance matrix equal to the identity matrix. Thus, based on the multivariate delta method, the variance of the percent recovery is given by(AI.4)

where is the gradient of . Using the gradient of *Q*_*p *_results of the quality control procedure (standard deviations of the percentages of recovery for PFAs in ppb concentrations), and by taking *c*_*p *_as the sample average of the ppb concentrations recorded for exposure across-sample, estimates for the measurement error variability for each exposure can be obtained as follows

Using that information, the estimate of covariance matrix *∑* in (2) is given by(AII.1)

## Appendix II. Justification for conditional likelihood in matched case-control studies with measurement error in continuous exposures

Bayesian justifications for using conditional likelihood when actual exposure is observed are given by Rice [11,22,23], but the situation is less clear when the actual exposure is unobserved and treated as an unknown quantity inside the posterior distribution. Thus we provide the following argument for using the conditional likelihood as a disease model, as long as the model for exposure acknowledges both across-stratum and within-stratum variation. For simplicity the argument is presented in the situation without confounders that vary within matched sets, i.e., all confounding is addressed via matching.

Under the Bayesian paradigm, for individually matched case-control data retrospectively collected and subject to measurement error, the joint posterior model of the true exposure and surrogate variables for a specific stratum (matched set) *s *is given by

The first term of the right hand side of (AII.1) is obtained under the assumption of *nondifferential *measurement error model. Regarding the second term, since  depends explicitly on the values of the elements of the vector *Y*, the distribution of (*X, Y*= (1,0, ..., 0), *S*) is the same as the distribution of . Therefore, the retrospective probability in the second term of the right hand side of (AII.1) can be rewritten as(AII.2)

Notice the distribution of *Y*is independent of the specific stratum, and it is mainly determined by the exposure variables. Therefore, by standard arguments, under a prospective logistic regression model with stratum-specific intercept, the probability of  is simply the conditional likelihood term. On the other hand, arguably the dominant variation in the prospective distribution of  given (*X, S*) will be with *S *rather than *X*, via the stratum-specific intercept. As a result, . Thus, (AII.2) can be approximated as

Since  are *exchangeable*, it is possible to see that

Thus, the joint posterior density of the true exposure and surrogate variables for a specific stratum *s *can be expressed as(AII.3)

where the conditional density of (*W*|*X*) corresponds to the measurement model. This describes how the surrogate vector of explanatory variables arises from the true values of *X*. And the density (*X*|*S*) of refers to the within-stratum density of the exposure model. The within-stratum density of the exposures can be implemented as a random-effect model. Therefore, the use of a conditional logistic regression model, when the exposures are measured with error, is justified by having a random-effect exposure model.

## Pre-publication history

The pre-publication history for this paper can be accessed here:

http://www.biomedcentral.com/1471-2288/11/67/prepub

## Supplementary Material

Additional file 1 **WinBUGS Code**. Code used to perform the Bayesian adjustment for measurement error in a matched case-control study with multiple continuous covariates Click here for file
