# Supplementary material for: Bayesian adjustment for measurement error in continuous exposures in an individually matched case-control study
Source: BMC Med Res Methodol. 2011 May 14;11:67. doi: 10.1186/1471-2288-11-67 (PMC3120807; doi:10.1186/1471-2288-11-67)
Supplement: Additional file 1 — WinBUGS Code. Code used to perform the Bayesian adjustment for measurement error in a matched case-control study with multiple continuous covariates [file 1471-2288-11-67-S1.PDF]

Additional file 1

WinBUGS code for implementing the proposed Bayesian model to correct for measurement error in continues exposures

Simple model under a measurement error analysis (ME-S)

```
model {

	# HYPERPARAMETERS
	prec.mbar[1:3, 1:3]  <- inverse(var.mbar[,])
	prec.beta[1:3, 1:3]  <- inverse(var.beta[,])
	Nu0 <- K+2
	prec[1:3, 1:3] <- inverse(Var.measure[,])

	for(i in 1:N) {
	# MEASUREMENT ERROR MODEL
		for( j in 1:n[i]) {
				W[i, j, 1:3]  ~ dmnorm ( X[i, j, 1:3 ] , prec[,] )

	# RANDOM EFFECT EXPOSURE MODEL
				X[i, j, 1:3 ]  ~ dmnorm ( m[i, 1:3] , VwINV[ , ] )
				}
		 m[i, 1:3]  ~ dmnorm ( mbar[ 1:3] , VbINV[ , ] )

	# DISEASE MODEL: CONDITIONAL LIKELIHOOD
		Y[i, 1:n[i] ] ~ dmulti( p[i, 1:n[i] ], 1)
		 for (j in 1:n[i]) {
				p[i, j] <- e[i, j] / sum(e[i, 1:n[i]])
				log( e[i, j] )<- inprod(beta[1:3] , X[i, j, 1:3 ])
				}
		}

	# PRIORS
	mbar[1:3] ~ dmnorm( mu.mbar[], prec.mbar[,])
	beta[1:3] ~ dmnorm( mu.beta[], prec.beta[,])
	VwINV[1:3 , 1:3] ~ dwish(R0[,], Nu0)
	VbINV[1:3 , 1:3 ] ~ dwish(R0[,], Nu0)

}
```

Adjusted model by confounders under a measurement error analysis (ME-A)

```
model {

	# HYPERPARAMETERS
	prec.mbar[1:3, 1:3]  <- inverse(var.mbar[,])
	prec.beta[1:3, 1:3]  <- inverse(var.beta[,])
	prec.betaC[1:4, 1:4]  <- inverse(var.betaC[,])
	Nu0 <- K+2
	prec[1:3, 1:3] <- inverse(Var.measure[,])

	for(i in 1:N) {
	# MEASUREMENT ERROR MODEL
		for( j in 1:n[i]) {
				W[i, j, 1:3]  ~ dmnorm ( X[i, j, 1:3 ] , prec[,] )

	# RANDOM EFFECT EXPOSURE MODEL
				X[i, j, 1:3 ]  ~ dmnorm ( m[i, 1:3] , VwINV[ , ] )
				}
		m[i, 1:3]  ~ dmnorm ( mbar[ 1:3] , VbINV[ , ] )

	# DISEASE MODEL: CONDITIONAL LIKELIHOOD.
		Y[i, 1:n[i] ] ~ dmulti( p[i, 1:n[i] ], 1)
		for (j in 1:n[i]) {
				p[i, j] <- e[i, j] / sum(e[i, 1:n[i]])
				log( e[i, j] )<-  (inprod(beta[1:3] , X[i, j, 1:3 ])  +
						inprod(betaC[1:4] , Z[i, j, 1:4 ]) )
				}
		}

	# PRIORS
	mbar[1:3] ~ dmnorm( mu.mbar[], prec.mbar[,])
	beta[1:3] ~ dmnorm( mu.beta[], prec.beta[,])
	betaC[1:4] ~ dmnorm( mu.betaC[], prec.betaC[,])

	VwINV[1:3 , 1:3] ~ dwish(R0[,], Nu0)
	VbINV[1:3 , 1:3 ] ~ dwish(R0[,], Nu0)

}
```

WinBUGS code for implementing the Bayesian *naive* analysis

Simple model under the naive analysis (N-S)

```
model {

	# HYPERPARAMETERS
	prec.beta[1:3, 1:3]  <- inverse(var.beta[,])

	# DISEASE MODEL: CONDITIONAL LIKELIHOOD
	for(i in 1:N) {
		Y[i, 1:n[i] ] ~ dmulti( p[i, 1:n[i] ], 1)
		for (j in 1:n[i]) {
				p[i, j] <- e[i, j] / sum(e[i, 1:n[i]])
				log( e[i, j] )<-  inprod(beta[1:3] , W[i, j, 1:3 ])
				}
		}

	# PRIORS
	beta[1:3] ~ dmnorm( mu.beta[], prec.beta[,])
}
```

Adjusted model by confounders under a naive analysis (N-A)

```
model {

	# HYPERPARAMETERS
	prec.beta[1:3, 1:3]  <- inverse(var.beta[,])
	prec.betaC[1:4, 1:4]  <- inverse(var.betaC[,])

	# DISEASE MODEL: CONDITIONAL LIKELIHOOD.
	for(i in 1:N) {
		Y[i, 1:n[i] ] ~ dmulti( p[i, 1:n[i] ], 1)

		for (j in 1:n[i]) {
				p[i, j] <- e[i, j] / sum(e[i, 1:n[i]])
				log( e[i, j] )<-  (inprod(beta[1:3] , W[i, j, 1:3 ])  +
						inprod(betaC[1:4] , Z[i, j, 1:4 ]) )
				}
		}

	# PRIORS
	beta[1:3] ~ dmnorm( mu.beta[], prec.beta[,])
	betaC[1:4] ~ dmnorm( mu.betaC[], prec.betaC[,])

}
```
